# Antimicrobial Evaluation of Various Honey Types against Carbapenemase-Producing Gram-Negative Clinical Isolates

**DOI:** 10.3390/antibiotics11030422

**Published:** 2022-03-21

**Authors:** Elisavet Stavropoulou, Chrysoula (Chrysa) Voidarou, Georgios Rozos, Natalia Vaou, Michael Bardanis, Theodoros Konstantinidis, Georgia Vrioni, Athanasios Tsakris

**Affiliations:** 1 Department of Microbiology, Medical School, National Kapodistrian University of Athens, 11527 Athens, Greece; gvrioni@med.uoa.gr (G.V.); atsakris@med.uoa.gr (A.T.); 2 Centre Hospitalier Universitaire Vaudois (CHUV), 1101 Lausanne, Switzerland; 3 Department of Agriculture, School of Agriculture, University of Ioannina, 47100 Arta, Greece; xvoidarou@uoi.gr (C.V.); clevervet@hotmail.com (G.R.); 4 Laboratory of Hygiene and Environmental Protection, Department of Medicine, Democritus University of Thrace, Dragana, 68100 Alexandroupolis, Greece; nvaou@hotmail.com (N.V.); m@ivm.gr (M.B.); tconstan@med.duth.gr (T.K.); 5 Gourmeli., 73100 Chania, Crete, Greece

**Keywords:** antimicrobial activity, physicochemical, honey, alternative, treatment, Gram-negative, VIM carbapenemase, KPC carbapenemase

## Abstract

The development of antibiotic resistance is a major public health issue, as infections are increasingly unresponsive to antibiotics. Emerging antimicrobial resistance has raised researchers’ interest in the development of alternative strategies using natural compounds with antibacterial activity, like honey, which has emerged as an agent to treat several infections and wound injuries. Nevertheless, the antibacterial effect of honey was mostly evaluated against Gram-positive bacteria. Hence, the objective of our study was to evaluate the antibacterial activity, as well as the physicochemical parameters, of genuine Greek honeys against multidrug-resistant Gram-negative pathogenic bacteria. In this vein, we aimed to study the in vitro antibacterial potential of rare Greek honeys against Verona integron-encoded metallo-β-lactamase (VIM)- or *Klebsiella pneumoniae* carbapenemase-producing multidrug-resistant Gram-negative pathogens. Physicochemical parameters such as pH, hydrogen peroxide, free acidity, lactonic acid, total phenols total flavonoids, free radical scavenging activities, tyrosinase enzyme inhibitory activity and kojic acid were examined. Moreover, the antimicrobial activity of 10 different honey types was evaluated in five consecutive dilutions (75%, 50%, 25%, 12.5% and 6.25%) against the clinical isolates by the well diffusion method, as well as by the determination of the minimum inhibition concentration after the addition of catalase and protease. Almost all the physicochemical parameters varied significantly among the different honeys. Fir and manuka honey showed the highest values in pH and H_2_O_2_, while the free acidity and lactonic acid levels were higher in chestnut honey. Total phenols, total flavonoids and free radical scavenging activities were found higher in cotton, arbutus and manuka honey, and finally, manuka and oregano honeys showed higher tyrosinase inhibition activity and kojic acid levels. The antimicrobial susceptibility depended on the type of honey, on its dilution, on the treatment methodology and on the microorganism. Arbutus honey was the most potent against VIM-producing *Enterobacter cloacae* subsp. *dissolvens* in 75% concentration, while fir honey was more lethal for the same microorganism in the 25% concentration. Many honeys outperformed manuka honey in their antibacterial potency. It is of interest that, for any given concentration in the well diffusion method and for any given type of honey, significant differences were not detected among the four multidrug-resistant pathogens, which explains that the damaging effect to the bacterial cells was the same regardless of the bacterial species or strain. Although the antimicrobial potency of different honey varieties dependents on their geographical origin and on their compositional differences, the exact underlying mechanism remains yet unclear.

## 1. Introduction

Antibiotics are used as prophylactic or therapeutic agents to treat human infections. Definitively, their proper clinical use increased life expectancy and reduced mortality. However, the misuse of antibiotics has led to the emergence of multidrug-resistant bacterial strains [1]. Under selective drug pressure, susceptible to antibiotics bacteria are killed or inhibited, while resistant bacteria or bacteria having acquired antibiotic-resistant characteristics have more chances to prevail [1,2].

The World Economic Forum reports antibiotic resistance as a global risk and one of the greatest threats to human health [3,4]. Moreover, antibiotic resistance leads to increasing health costs, extension of hospital stays and occasionally death. ECDC reports have shown that, over the last few years, a notable increase of combined resistance to multiple drugs has been observed [5]. As reported, an increase of antibiotic resistance in Europe, specifically by multidrug-resistant Gram-negative bacteria, has been observed [5]. The prescription and administration of major last-line antibiotics, such as carbapenems, has significantly increased as an aftermath of the rising multidrug resistance of Gram-negative bacteria. Recently, research data from the European Antimicrobial Resistance Surveillance Network (EARS-Net) showed that more than 33,000 people die each year in Europe from antibiotic-resistant infections [6,7]. Resistant bacteria in bearing humans are able to spread in every environment, such as water, food, air, plants and animals, creating “dynamic islands” of resistance spreading and vice versa. Yet, mobile resistance genes can be horizontally transmitted through bacteria of the same or different species [8]. Genes found on mobile elements such as plasmids and transposons encode the production of various carbapenemases, including *Klebsiella pneumoniae* carbapenemases (KPCs), and metallo-β-lactamases (MBLs), such as Verona integron-encoded metallo-β-lactamases (VIMs). KPC and VIM carbapenemases pose a serious threat in human health and particularly in health institution environments [9,10]. Facing this major issue, researchers have focused on the development of a new generation of antimicrobials, as well as on the deployment of alternate therapies to promote health and reduce infection risk, which will undoubtedly help to reduce the selective pressure that eventually leads to the emergence and transmission of antibiotic resistance genes.

Alternatives to antibiotics for the treatment of infectious diseases include bacteriophages [11,12], probiotics [13], bacteriocins [14] and different natural products [15]. Since ancient times, honey has been traditionally used as a remedy, as shown in Sumerian and Babylonian cuneiform writings; the Hittite code and the sacred writings of India, China [16] and Egypt dating back to 2100–200 BC [17]. Aristotle (384–322 BC) reported honey as being “good as a salve for sore eyes and wounds”. Even from these ancient reports, honey seemed to be effective in bacterial infections, gastrointestinal diseases, allergic and immunologic disorders and traumatic wounds [16]. 

Honey is a natural concentrated sweetener containing basically the monosaccharides fructose and glucose. It is produced by bees that collect floral nectar or honeydew to support the metabolism of flight muscles during foraging or storage as food supply. While floral honey is made from blossoms nectar, honeydew is made from the secretions of plant living parts or even the excretions of plant-sucking insects [18]. The most common honey medical use is topical wound healing activity, as it preserves wound moistness due to its viscosity that acts as a barrier to infection [17]. Immunomodulatory, antioxidant and antimicrobial activities have been found in different honeys [19,20,21,22,23,24]. Obviously, different botanical origins and geographical locations around the world where honey is collected influence its characteristics and activities. Compounds found in honey [19], such as hydrogen peroxide, polyphenols, methylglyoxal and bee-defensin 1, seem to be related to the antimicrobial potency. Vitamins are also found in honey, including pantothenic acid, niacin, ascorbic acid and riboflavin, as well as minerals such as iron, magnesium, manganese, potassium, zinc, ascorbic acid, manganese and phosphorus [25]. In the scientific literature, many reports have referred to the chemical profile of honey and the contribution of its components to nutrition [25]. Studies have shown that honeys can inhibit bacterial growth in different degrees according to their botanical origin and geographical location [26,27,28]. A unique antimicrobial activity was attributed to manuka honey, which gained much attention and was extensively studied [19,28]. Manuka honey was found effective against a wide range of pathogens [29]. However, it was found that different origins of manuka honey showed differing effects, and it seems to be less effective against Gram-negative bacteria, which are more resistant than Gram-positive bacteria [29]. Due to the fact that manuka honey showed interesting medicinal properties, it has been approved in combination with adjuvant treatments of wounds together with antimicrobial agents as a medical grade honey [29].

The antibacterial effect of honey was mostly evaluated against Gram-positive bacteria [30]. Thus, more research addressing the antibacterial activity of honey against Gram-negative bacteria and, specifically, against multidrug-resistant Gram-negative pathogenic bacteria is necessary. Such research should include a comparison of different types of honeys to detect their most potent biological actions as a therapeutical agent. The development of novel pharmaceutical substances that could inhibit KPC and VIM enzymes is compromised by the fast evolution of carbapenemases [31,32,33]. Therefore, our aim was to evaluate the antimicrobial activity of some rare genuine Greek honeys of different botanical origins in vitro against KPC- or VIM-producing multidrug-resistant Gram-negative clinical isolates as novel agents for therapeutic approaches against antibiotic-resistant bacteria based on their in vitro efficacy.

## 2. Results

Although bees graze various plants, usually beekeepers place hives in areas where there is a dominant plant species responsible for the flavor of the honey and consequently gives its name to the final product. The main physical and chemical parameters of the different types of Greek honeys are presented in Table 1 and Table 2. 

The pH values of the different honeys range from 3.22 ± 0.13 to 4.87 ± 0.21. The highest pH was found for fir honey, followed by manuka honey. The pH of fir honey was found significantly different with a significance level *p* < 0.05 comparing to cotton, arbutus, chestnut, thyme, orange, oregano, manuka and heath honeys.

The hydrogen peroxide values ranged from 0.36 ± 0.05 mM in sunflowers to 3.02 ± 0.05 mM for fir honey. The latter’s value was significantly different compared to any other honey’s hydrogen peroxide value and the only difference observed in this parameter. Besides fir honey, other honeys with high values of hydrogen peroxide originated from oregano, heath and manuka, while lower values showed in orange honey, thyme honey and cotton honey.

The results of the free acidity and lactonic acid for the honeys are reported in Table 1. There was significant difference in the values of free acidity and lactonic acid. The levels of lactonic acid ranged from 7.10 ± 0.20 meq kg^−1^ for manuka honey to a maximum of 15.44 ± 4.18 meq kg^−1^ for chestnut honey, while free acidity varied from 15.2 to 42.80 ± 14.01 meq kg^−1^ for chestnut honey.

The honeys were examined for the determination of the total phenols (TPC), total flavonoids (TFC) and their free radical scavenging activities (DPPH). TPC ranged from a maximum of 99.7 ± 39.89 mg GAE 100 g^−1^ for cotton honey to 30.6 ± 1.71 mg GAE 100 g^−1^ for sunflower honey. TFC ranged from 1.1 ± 0.18 for orange honey to a maximum of 4.22 ± 0.64 mg QE 100 g^−1^ for arbutus honey. The free radical scavenging power (DPPH) ranged from 0.9 ± 0.25 mg GAE kg^−1^ for orange honey to a maximum of 5.1 ± 0.8 mg GAE kg^−1^ for manuka honey. Significant differences were shown for fir, arbutus, chestnut, orange, oregano, sunflower and heath honey in phenolics, while sunflower honey showed significant differences with cotton, arbutus, chestnut, oregano and fir honey. Finally, free radical scavenging activity showed significant differences in manuka, cotton, arbutus, chestnut, thyme, orange, oregano, fir, sunflower and heath honey.

The tyrosinase enzyme inhibitory activity of the different honeys is shown in Table 2. The tyrosinase inhibition activity ranged from 29.40 ± 2.85 to 85.11 ± 4.33%. The highest activity of tyrosinase inhibition was found in manuka honey, followed by oregano honey (81.92 ± 4.90%), and the lowest inhibition activity was for sunflower honey.

Finally, the kojic acid results of the different honeys also appear in Table 2. The kojic acid levels ranged from 210.15 ± 12.26 mg koji kg^−1^ for manuka honey to 4.99 ± 1.12 mg koji kg^−1^ for sunflower honey.

Almost all parameters, except for lactonic acidity, varied significantly among the different honeys. However, in most cases, these parameters did not correlate with each other, as shown in Table 3. Yet, the observed significant correlations were all positive and moderate to strong (e.g., tyrosinase inhibition and total phenolic content, r = 0.636) or strong (e.g., lactonic acidity and free acidity, r = 0.952). 

**Figure 1:**
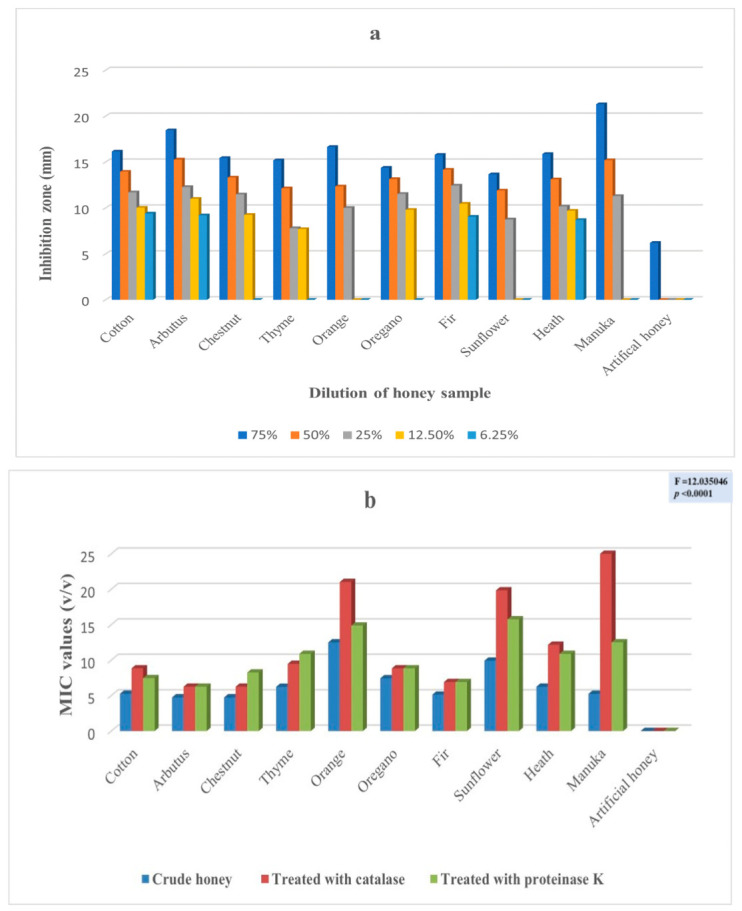
(**a**). Antibacterial activity (zone of inhibition in mm) of various concentrations of the different honeys against *Enterobacter cloacae* subsp. *dissolvens* assessed by the well diffusion method; (**b**). Geometrical means of the antibacterial activity of the different honeys against *Enterobacter cloacae* subsp. *dissolvens* assessed by the MIC method as the crude sample, with the addition of catalase and proteinase K.

**Figure 2:**
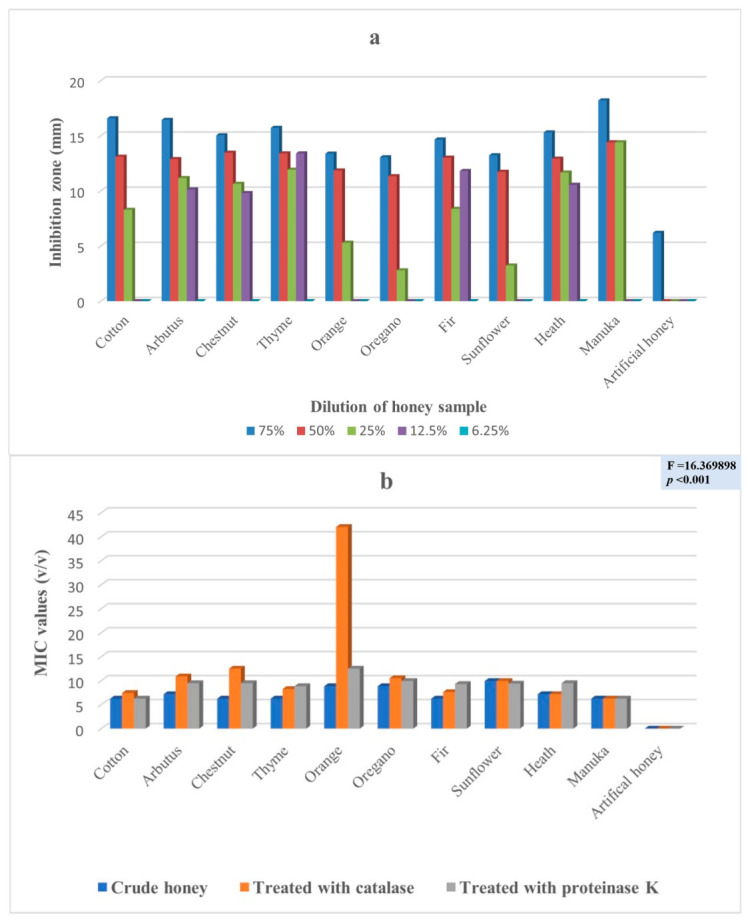
(**a**) Antibacterial activity (zone of inhibition in mm) of various concentrations of the different honeys against *Pseudomonas aeruginosa* assessed by the well diffusion method; (**b**) Geometrical means of the antibacterial activity of the different honeys against *Pseudomonas aeruginosa* assessed by the MIC method as the crude sample, with the addition of catalase and proteinase K.

**Figure 3:**
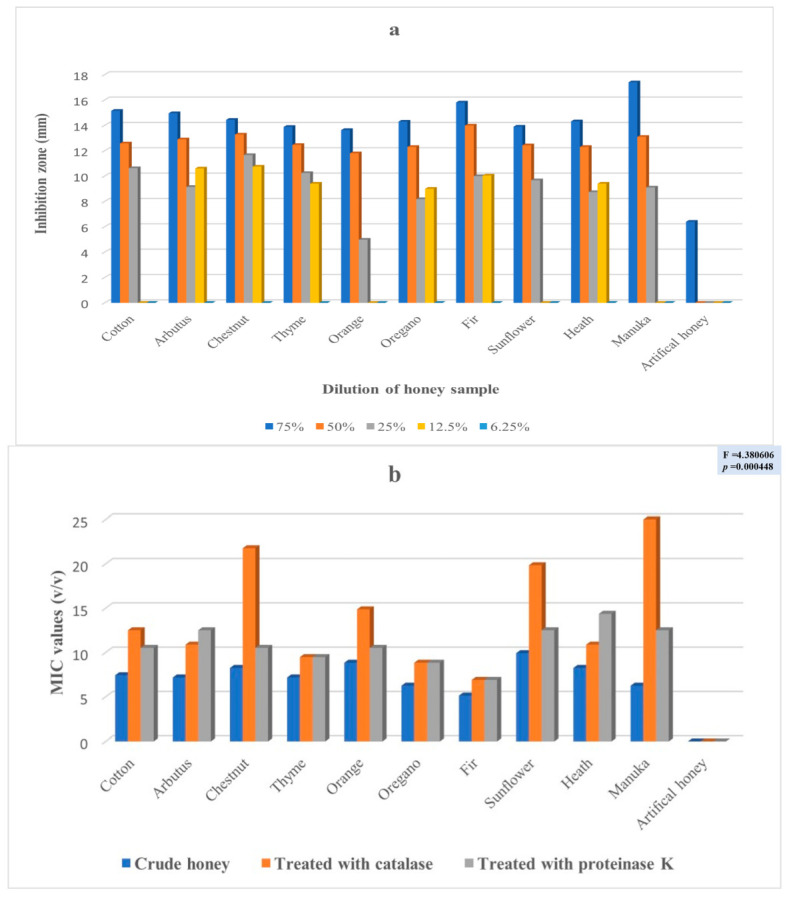
(**a**) Antibacterial activity (zone of inhibition in mm) of various concentrations of the different honeys against *Klebsiella pneumoniae* subsp. *pneumoniae* (1) assessed by the well diffusion method; (**b**) Geometrical means of the antibacterial activity of the different honeys against *Klebsiella pneumoniae* subsp. *pneumoniae* (1) assessed by the MIC method as the crude sample, with the addition of catalase and proteinase K.

**Figure 4:**
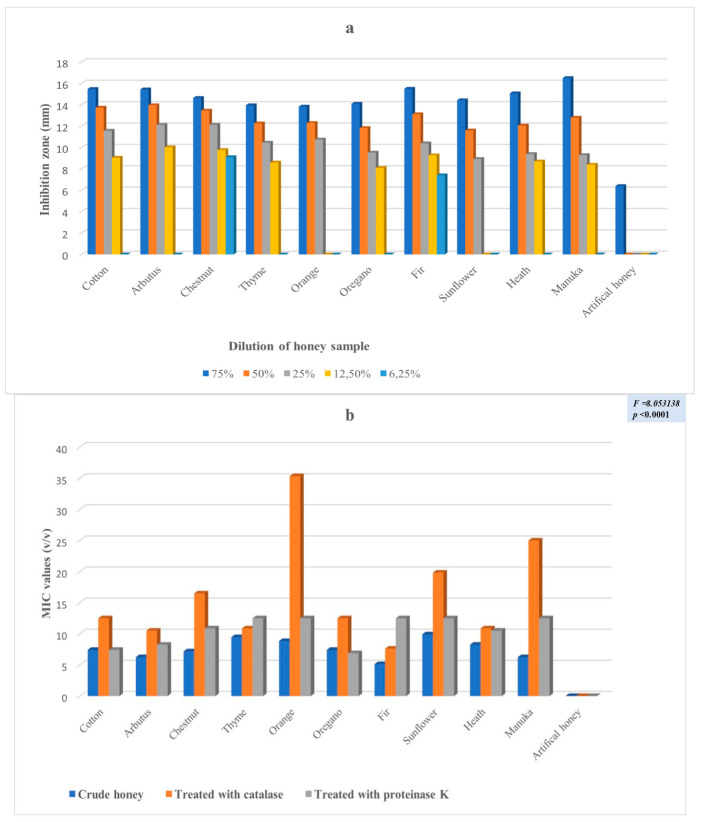
(**a**) Antibacterial activity (zone of inhibition in mm) of various concentrations of the different honeys against *Klebsiella pneumoniae* subsp. *pneumoniae* (2) assessed by the well diffusion method; (**b**) Geometrical means of the antibacterial activity of the different honeys against *Klebsiella pneumoniae* subsp. *pneumoniae* (2) assessed by the MIC method as the crude sample, with the addition of catalase and proteinase K.

The antimicrobial potency of the honeys was assessed in five consecutive dilutions (75%, 50%, 25%, 12.5% and 6.25%) against four (4) clinical isolates by the well diffusion method, as well as by the determination of the minimum inhibition concentration. Figure 1a,b (Tables S1 and S2 in the Supplementary Material) presents the results for *Enterobacter cloacae* subsp. *dissolvens*. While Figure 2a,b (Tables S3 and S4 in the Supplementary Material), Figure 3a,b (Tables S5 and S6 in the Supplementary Material), and Figure 4a,b (Tables S7 and S8 in the Supplementary Material) show the results for *Pseudomonas aeruginosa* and for the two (2) strains of *Klebsiella pneumoniae*, respectively. These results show a complicated picture, because the observed susceptibility depended on the type of honey, on the concentration or dilution (well diffusion method), on the treatment of the honey (MIC assessment) and on the microorganism. For example, arbutus honey was the most potent in the well diffusion method against *E. cloacae* subsp. *dissolvens* in the 75% concentration, while fir honey was more lethal for the same microorganism in the 25% concentration. As far as the MIC assessment is concerned, statistically significant differences were recorded only in the samples in which catalase was added. It should be noted that, as shown to Tables S1, S3, S5 and S7, the manuka sample did not show any inhibition zone at concentrations 12.5% and 6.25% (well diffusion assay), while many samples of the Greek honeys exerted antibacterial activity at these concentrations.

Another interesting finding was that, for any given concentration in the well diffusion method, and for any given type of honey, there were not any significant differences (one-way ANOVA for *p* < 0.005) among the four isolates, meaning that the destroying effect to the bacterial cells was the same regardless of the species or of the strain. 

Manuka honey is well-known for its antibacterial activity and was incorporated in the present study as a criterion for the antibacterial potency of the Greek honeys. Table 4 and Table 5 show the number of samples that outperformed the antibacterial effect of manuka honey, regardless of their phytological origin, for the well diffusion method and the MIC assessment, respectively. Quite a few samples outperformed manuka honey, a finding that confirms that the overall picture is an interaction of the type of honey, of the method, of the treatment of the sample and of the bacterial strain.

**Figure 5:**
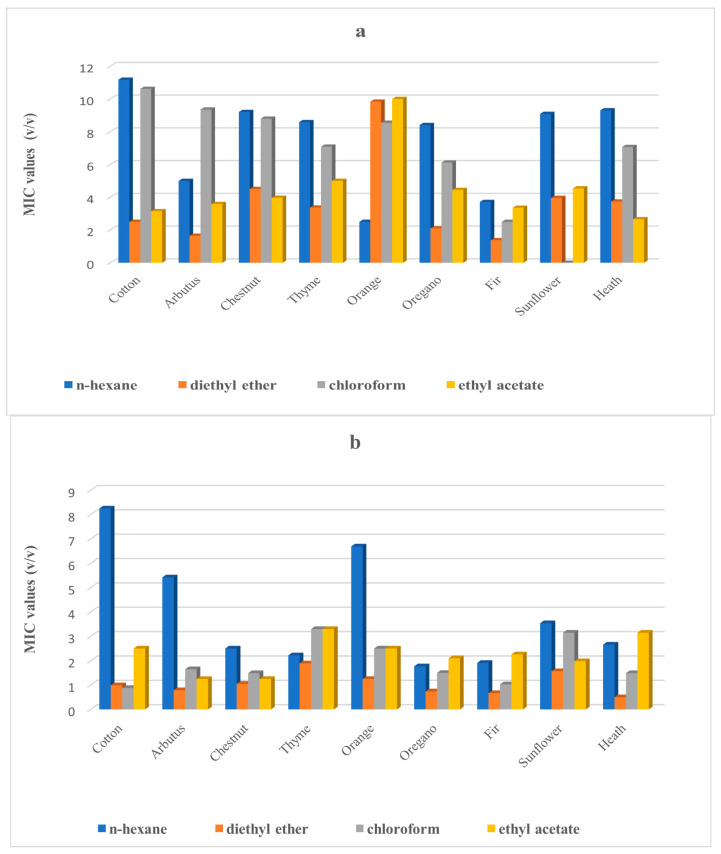

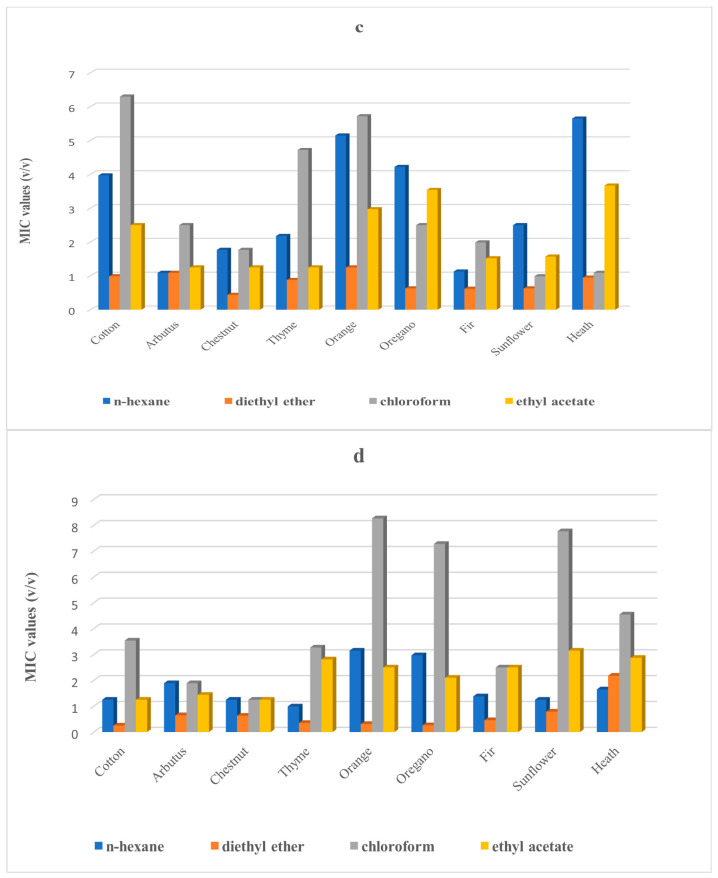
(**a**–**d**) Geometrical means of the MICs values of the honey extracts by different solvents; (**a**) *Pseudomonas aeruginosa*, (**b**) *Klebsiella pneumoniae* subsp. *pneumoniae* (1), (**c**) *Enterobacter cloacae* subsp. *dissolvens* and (**d**) *Klebsiella pneumoniae* subsp. *pneumoniae* (2), respectively.

Figure 5a–d (Tables S9–S12 in the Supplementary Material), show the antibacterial activity of the extracts of honey samples after treatment with four different organic solvents. The assessment of this activity was performed by the MIC method based on the principle that the different solvents can extract different mixtures of compounds and could, to some extent, clarify the antibacterial activity of the honeys. The results showed that the different extracts had different impacts on the bacterial growth, although these effects were also species or strain-specific. In the case of *P. aeruginosa*, the honey samples for each solvent showed significant differences in their antibacterial effects, while, in the case of *Klebsiella pneumoniae* subsp. *pneumoniae* (1), only the n-hexane extracts showed differentiation of the honey samples in the growth inhibition effect. All solvents extracts, except ethyl acetate, had significantly different impacts on the growth of *K. pneumoniae* subsp. *pneumoniae* (2). Finally, the n-hexane and ethyl acetate extracts inhibited significantly the growth of *E. cloacae* subsp. *dissolvens*. A critical observation is that the different solvents enhance or reduce the antibacterial effects of the same honey sample. For example, cotton honey’s n-hexane solvent shows the highest MIC, while the same honey’s extra by diethyl ether MIC is numerically low with respect to the other honey MICs in the same solvent.

## 3. Discussion

Honey has been reported early in human writings. *Homo Sapiens* have eaten honey since the Stone Age. Traditional medicines among which honey has been applied to treat infections have existed since the origin of mankind [16]. Honey has shown a broad spectrum of antimicrobial activity [34] against common wound pathogens [35]. Moreover, it was shown to be effective against antibiotic-resistant bacteria and strengthen the efficacy of several antibiotics against resistant bacteria [21,36]. Yet, honey was found to be effective against not only aerobes but anaerobes as well [22]. Although there is a plethora of works on the evaluation of the antimicrobial activity of honey, most have focused on Gram-positive bacteria, and less research has been done against Gram-negative bacteria [37,38]. Notably, a lot of research efforts have been oriented towards the effect of manuka honey against methicillin-resistant *Staphylococcus aureus* (MRSA) in vitro [26,39,40,41,42].

In this context, the aim of this study was to investigate the in vitro antimicrobial activity of honeys against Gram-negative bacteria. In our study, we enrolled four genotypically confirmed carbapenemase-positive clinical strains: (1) VIM-producing *P. aeruginosa*, (2) KPC-producing *K. pneumoniae*, (3) VIM-producing *E. cloacae* subsp. *dissolvens* and (4) VIM-producing *K. pneumoniae*. These strains were isolated from bloodstream infections in hospitalized patients of a tertiary care university hospital located in the region of Attica (Greece) and belong to the Bacterial Collection of the Department of Microbiology, Medical School, National and Kapodistrian University of Athens. KPC and VIM-bearing bacteria have caused severe syndromes in patients in Greece, Italy and Spain [9]. Due to its antimicrobial activity, manuka honey has the ability to strengthen the efficacy of several antibiotic drugs [36]. Many studies have demonstrated its antimicrobial activity against multidrug-resistant microorganisms [43]. However, this effect seems to be more potent against Gram-positive than Gram-negative bacteria [37,44,45].

**Figure 6:**
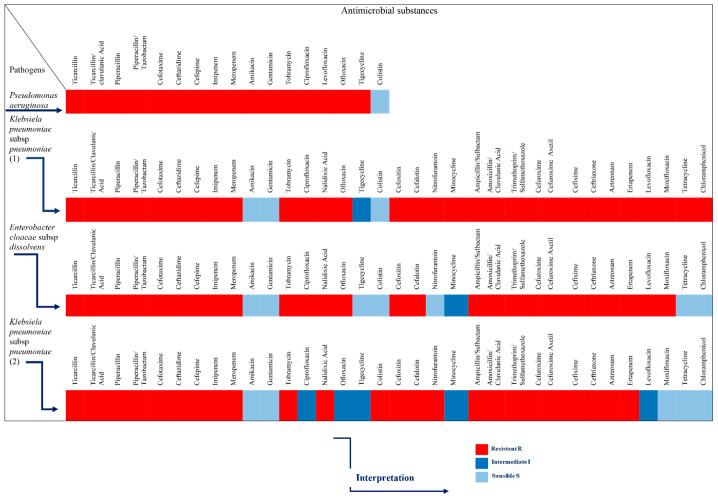
Antibiotic susceptibility pattern of the strains used in the study.

To experimentally investigate the antimicrobial potency of honey, our experimental design followed certain steps. To start, the VIM pathogens were identified, and their resistance to several antibiotics was verified (Figure 6). Then, these strains were exposed to different concentrations of the different honeys, which were first identified and analyzed for their physical and chemical characteristics. In this step, the antibacterial potency of the honeys was assessed by the well diffusion assay, measuring the diameter of the inhibition zone (Tables S1, S3, S5 and S7 and Figure 1a, Figure 2a, Figure 3a and Figure 4a). Additionally, the comparison among the different honeys was useful. Next, catalase and protease were added in the samples to neuter the antimicrobial activity of hydrogen peroxide and of various peptides and, thus, assess part of the antimicrobial potency of honeys due to these factors (Tables S2, S4, S6 and S8 and Figure 1b, Figure 2b, Figure 3b and Figure 4b). This assessment was performed by the MIC method. Finally, the samples of the different honeys were thoroughly mixed with organic solvents, and the recovered extracts were checked for their antibacterial activity against the clinical isolates. Different solvents extract different mixtures of compounds qualitatively or quantitatively, and these different mixtures were found to exert different antibacterial activities. Naturally, these issues cannot be exhausted by a series of in vitro testing like this experiment, but the authors believe that a sound foundation for further research has been laid.

The antibacterial activity of the different honeys is related to their various constituents and has different antimicrobial potential. Manuka honey antibacterial activity is attributed to the elevated levels of methylglyoxal (MGO) and was found particularly effective against antibiotic-resistant Gram-positive organisms such as MRSA [36]. Moreover, MGO as an active ingredient acts synergically with linezolid to strengthen its activity [36]. While manuka honey’s antimicrobial activity is related to MGO, other kinds of honeys seem to develop an antimicrobial activity due to the enzymatic production of hydrogen peroxide [17,36].

The antimicrobial effect and mechanism may be also related to the low pH of honey, as well as to the high sugar content that makes honey a high osmolarity product [17]. Honey is an acidic product due to the presence of organic acids, and its pH ranges usually between 3.2 and 4.5, which could directly inhibit several pathogenic bacteria [15], such as *Escherichia coli*, *Salmonella* spp., *P. aeruginosa* and *Streptococcus pyogenes,* having a pH value around 4 [17,46]. In our study, manuka honey revealed a pH mean value of 4.10 ± 0.15, hydrogen peroxide mean value 52.00 ± 2.74 µg/g, free acidity 15.2 ± 0.20, lactonic acidity 7.10 ± 0.20, TPC 88.71 ± 0.3 mg GAE/100 g, TFC 4.1 ± 0.80 CE/100 g), DPPH 5.1 ± 0.8, tyrosinase inhibition 85.11 ± 4.33% and kojic acid 210.15 ± 12.26 mg koji kg^−1^. As stated, due to its high sugar content, honey shows an important osmotic effect that, along with the low pH due to the presence of gluconic acid, contributes to its antibacterial property [47]. Honey acidic pH is the result of the conversion of glucose into gluconic acid by glucose oxidase, with hydrogen peroxide being a byproduct of the reaction [38].

The antimicrobial effect of the tested honeys was evaluated by five dilutions (75%, 50%, 25%, 12.5% and 6.25%) against the four multi-resistant clinical strains. The well diffusion assay (Table S1 and Figure 1a) showed arbutus honey to be most potent against *E. cloacae* subsp. *dissolvens* at a 75% concentration, while fir honey was more lethal against *E. cloacae* subsp. *dissolvens* at a 25% concentration. Concerning, manuka honey, no inhibition was observed at concentrations 12.5% and 6.25%, while many samples of the Greek honeys showed antibacterial effects at these concentrations, such as cotton, arbutus, fir and heath at 6.25% and cotton, arbutus, chestnut, thyme, oregano, fir and heath at 12.5% concentrations (Table S1 and Figure 1a). Almost all studied honeys exhibited antimicrobial activity at 25% and 50% concentrations, notwithstanding this effect was more pronounced at the 75% concentration, ranging from a maximum of 21.3 mm to a minimum of 13.67 mm (Table S1 and Figure 1a). Artificial honey showed a slight antibacterial effect (6.2 mm) only at a 75% concentration (Table S1).

The geometrical means of the antibacterial activity of the different honeys against all four clinical isolates obtained by the MIC assessment method showed statistically significant differences notably in honeys when catalase was added (Tables S2, S4, S6 and S8 and Figure 1b, Figure 2b, Figure 3b and Figure 4b), implying that the hydrogen peroxide content is a key factor. The fact that the average values of hydrogen peroxide of the samples were not significantly different (except for fir, Table 1) suggests that, besides its direct antibacterial activity, hydrogen peroxide may be involved in the pathways leading to antibacterial compounds, explaining the differences when catalase was added.

The antibacterial activity of various concentrations of the different honeys against *P. aeruginosa* assessed by the well diffusion method showed an effect at 6.25%, while only five honeys (arbutus, chestnut, thyme, fir and heath) exhibited a low effect at a 12.5% concentration (Table S3 and Figure 2a). All studied honeys showed an antimicrobial effect at a 50% concentration, ranging from a maximum of 14.4 mm (manuka honey) to a minimum of 13.67 mm (Table S3 and Figure 2a). The most potent antimicrobial effect was at a 75% concentration, as shown by manuka honey (18.2 mm), followed by cotton honey (16.58 mm) and arbutus (16.44%). The MIC assessment for *P. aeruginosa* showed statistically significant differences notably in honeys with catalase added (Table S4 and Figure 2b).

Concerning the *K. pneumoniae* subsp. *pneumoniae* (1) strain, the well diffusion assay showed no inhibition 6.25% for the different honeys. At a 12.5% concentration, six honeys (arbutus, chestnut, thyme, oregano, fir and heath) showed limited antimicrobial activity, while all of them showed an antimicrobial effect at a 25% concentration (Table S5 and Figure 3a). The antimicrobial activities of the different honeys at 50% against *K. pneumoniae* subsp. *pneumoniae* (1) were considerable, while more potent action was exhibited at a 75% concentration for almost all honeys, ranging from a maximum of 17.40 mm (manuka honey), followed by 15.81 mm for fir and a minimum of 13.63 mm for orange honey (Table S5 and Figure 3a). Artificial honey showed a slight inhibition at the 75% concentration only. Finally, the MIC assessment for *K. pneumoniae* subsp. *pneumoniae* (1) showed statistically significant differences in honeys when catalase was added (Table S6 and Figure 3b).

The well diffusion assay at a 6.25% concentration exhibited low inhibition zones against *K. pneumoniae* subsp. *pneumoniae* (2) for chestnut and fir (Table S7 and Figure 4a). At a 12.5% concentration, almost all honeys showed inhibition zones, except orange and sunflower (Table S7 and Figure 4a), while all of them exhibited an antimicrobial effect at 25%, ranging from a minimum of 9.3 mm (manuka honey) to the maximum of 12.13 mm for arbutus and chestnut honey (Table S7 and Figure 4a). Increasing values were observed for all honeys at the 50% concentration, while a 75% concentration registered values from 16.5 mm for manuka honey, followed by fir (15.50 mm), cotton (15.48 mm) and arbutus (15.44 mm). It is of note that manuka honey showed a smaller effect than most of the other honeys at low concentrations and exhibited the most potent effect at 75% (Table S7). Similarly, the MIC assessment for *K. pneumoniae* subsp. *pneumoniae* (2) showed statistically significant differences in honeys with catalase added (Table S8 and Figure 4b).

Many honey samples were found to outperform manuka honey’s antibacterial activity in the well diffusion assay (chi-square, statistical significance level *p* < 0.05). An analysis revealed that, in the lower concentrations, more samples outperformed the manuka honey than in higher ones (Table 4), a finding suggesting that the antibacterial factors of this honey are less effective when the honey is diluted, while the antibacterial factors of the Greek honeys are more drastic. Manuka honey antibacterial activity has been extensively studied [43,48,49]. Thus, we enrolled manuka honey in our study as a comparative criterion for the antibacterial potency of the studied honeys. Several Greek honeys included in our study outperformed the antibacterial effect of manuka honey (Table 4 and Table 5), regardless of their phytological origin. Arbutus, chestnut and fir honeys seemed to exhibit a more potent antimicrobial effect against our Gram-multi-resistant strains.

As far as the MIC assessment method is concerned, the same conclusion is valid, that the antibacterial agents of manuka honey function effectively in the crude environment while the antibacterial factors of the Greek honeys function better when catalase and protease are added (chi-square, statistical significance level *p* < 0.05, Table 5). 

It is of interest that, for any given concentration in the well diffusion assay and for any given type of honey, there were not any significant differences (*p* < 0.005) among the four multi-resistant clinical strains, meaning that the destroying effect to the bacterial cells was similar independent of the bacterial species or strain tested (Table 4 and Table 5). No strain was found more vulnerable than another. A possible explanation is that the antibacterial substances of the honeys kill Gram-negative bacterial cells in the same way regardless of the species (as far as the three species tested are concerned).

The correlation between the physicochemical parameters of the honeys (Spearman’s rho coefficient, statistical significance for *p* < 0.05, Table 3) showed that most of these characteristics were independent. It is not a surprise that the free acidity correlated positively to the lactonic acidity, since both types of acidity originate from the metabolism of carbohydrates. The correlation of kojic acid to lactonic acidity would be a reasonable expectation, since kojic acid is produced from carbohydrates, with lactone being an intermediate product [49,50], but it was not observed. 

Antibacterial properties of honey have been attributed to different physicochemical factors, such as acidity, osmotic effect and high sugar concentration but also to the presence of bactericidal compounds such as hydrogen peroxide; various antioxidants and enzymes such as lysozyme, polyphenols and flavonoids [50,51].

Honey acidity imparts important chemical and sensorial characteristics and thus contributes to its safe storage [50,51]. As is well-known, gluconic acid is the major organic acid found in honey derived from an enzymatic oxidation following the interaction of the enzyme’s glucose oxidase with glucose in the bee’s stomach [25]. The honey pH acidity [52,53] upon digestion dilution by gastric acid in the stomach activates glucose oxidase that releases hydrogen peroxide, which has an important antibacterial activity [49,50]. However, the process of gluconic acid formation is multifactorial and depends on the concentrations of the enzymes and glucose, as well as on the oxygen supply, the pH value and the temperature [54,55,56,57]. Yet, hydrogen peroxide stimulates the fibroblasts and the epithelial cells involved in the healing procedure of wounds [51,52]. High levels of hydrogen peroxide are related to high levels of glucose oxidase, but also, the action of catalase is related to hydrogen peroxide, with an inverted relation [58]. In this vein, it was hypothesized that the antibacterial action of honey could be entirely dissociated by the addition of catalase [58,59] that removes H_2_O_2_, although other compounds seem to be involved in the antibacterial activity of honey [59].

Organic acids are responsible for the acidic properties of honey [60]. The various honey pH in our study ranged from pH 3.22 (heath honey) followed by 3.23 (oregano) to pH 4.87 (fir honey), while manuka honey showed a pH of 4.10 (Table 1). It must be mentioned that honey acidity is an important factor contributing to its antimicrobial potency, since bacterial growth is usually effective at higher pHs ranging between 6.5 and 7.5. Yet, as stated, the antimicrobial activity in several honeys depends on the hydrogen peroxide content. Honey produces hydrogen peroxide when it is diluted, such as, when starting the conversion process of glucose in honey, the glucose oxidase requires a pH of 5.5–8.0 In our study, the H_2_O_2_ presence was determined by measuring the levels of H_2_O_2_ in a dilution of 40% before and after its removal by catalase. Fir honey showed a higher H_2_O_2_ (3.02 mM) compared to more acidic heath and oregano honeys, which showed H_2_O_2_ concentrations of 1.29 mM and outreached manuka honey (Table 1). Low oxygen peroxide levels were observed in sunflower and orange (0.36 mM) honeys, followed by thyme (0.59 mM) (Table 1). The authors stated [61] that hydrogen peroxide can increase in honey constantly during prolonged incubation and diluted honey. It must be mentioned that acidic (low pH) is effective as an antibacterial factor in undiluted honey.

Free acidity is measured in the presence of hydrolysable ions [62]. Free acidity is the excess acidity of stoichiometrically balanced salts and was determined as honey alone does not contain enough sodium salts. The lactonic acid values were also determined. It is interesting that, although free acidity and lactonic acidity showed a strong positive correlation (r = 0.952), lactonic acidity did not show any significantly different values among the types of honey, while free acidity did. Therapeutic approaches were attributed to free acidity and lactonic acid [63,64]. Nevertheless, they have biological activities and diverge in action, as free acidity is associated with a higher virulence and proinflammatory cytokine activity, while lactonic acid activity has strong spermicidal and anticancer activity [64]. Higher free acidity (42.80 meq kg^−1^) and lactonic acid (15.44 meq kg^−1^) are reported for chestnut honey, followed by arbutus and oregano honey (Table 1). These values outreached considerably manuka honey (free acidity, 15.2 meq kg^−1^; lactonic acid, 7.10 meq kg^−1^) (Table 1).

The antibacterial action of honey is owed to its osmotic action and high sugar concentration [24], which was checked by comparison to artificial honey in all five concentrations used (75% *v*/*v*, 50% *v*/*v*, 25% *v*/*v*, 12.5% *v*/*v* and 6% *v*/*v*). It showed no significant inhibitory action, which could support an argument claiming that the hyperosmotic effect is mainly responsible for the antibacterial action. The diameters zones in the well diffusion assays were smaller the more diluted the honey was. It is believed that the presence of other chemical compounds that are equally diluted could impact upon the presence of smaller inhibition zones [24].

As previously discussed, MGO has been found as the key component for the non-peroxide activity exhibited by manuka honey [34].

Honey contains very small quantities of proteins and peptides in the vicinity of 0.1–0.5%, with molecular weight from 20 kDa to 80 kDa [65]. In recent studies, the presence of an antimicrobial peptide in honey “bee defensin-1” has been demonstrated to be an important antimicrobial factor in honey [53]. However, in manuka honey, no evidence of antimicrobial peptides was found [54]. Although bee defensin-1 is effective mainly against Gram-positive bacteria, some studies performed with a recombinant version of defensin-1 demonstrated potency against Gram-negative bacteria, such as *P. aeruginosa and Salmonella choleraesuis* [66]. In our study the addition of protease did not induce any statistical difference in the antibacterial potency of the tested samples, perhaps due to the small amounts of proteins in all types of honey. 

Globally, little is known about honey’s mechanisms of action against bacteria. A recent study [55] reported the effects of honey samples on the membrane potential, membrane integrity and metabolic activity, which were assessed using different fluorochromes. Changes associated with membrane polarization and membrane integrity were found, and notable metabolic disruption was observed upon *S. aureus* [53]. Moreover, the potency of the antimicrobial effect seems to be dependent on the honey type, quality, compositional differences and concentrations.

Flavonoids seem to induce severe damage to the cytoplasmic membrane of the bacteria, leading to cell autolysis [56]. Similarly, the levels of specific and total phenolics analyzed identify p-coumaric acid, hesperetin and quercetin compounds [57]. These substances are metabolites of plants and originate from the nectar and are found in honey in quantities that depend on factors such as the floral variety of the area in which the bees graze, the geographical location, the time of the year and the storage conditions [67]. Quercetin was found to breach the membrane permeability, abrupt electrical potential and lower ATP synthesis [58]. Honey possesses antibacterial compounds with actions, such as β-lactamic antibiotics, antibacterial peptides or inhibitors of proton motive force [59]. The authors provided knowledge that honeys with enhanced antimicrobial activity showed high levels of total phenols (TPC), total flavonoids (TFC) and free radical scavenging activities (DPPH) [68]. Still, they claim that the addition of catalase to remove H_2_O_2_ impacts the antimicrobial activity of phenolics and H_2_O_2,_ in the chemical issue of mating these compounds and a mechanism based on the degradation of DNA by honey [59]. Polyphenols exported from honeys have the ability to degrade the plasmid DNA in the presence of H_2_O_2_ and Cu (II) in the Fenton-type reaction [59].

Our results show significant statistical differences in the total phenolic content and in the total flavonoid content among the various types of honeys. These differences can be attributed to the different floral origins and provide data that honeys with enhanced antimicrobial activity showed high levels of total phenols (TPC), total flavonoids (TFC) and free radical scavenging activities (DPPH) [68]. The addition of catalase to remove H_2_O_2_ impacts the combined antimicrobial activity of phenolics and H_2_O_2_ due to chemical synergy of these compounds and a mechanism based on the degradation of DNA by honey [59]. Polyphenols exported from honeys have the ability to degrade the plasmid DNA in the presence of H_2_O_2_ and Cu (II) in the Fenton-type reaction [59].

The total phenols (TPC) (Table 1) were found higher in cotton (99.7 mg GAE 100 g^−^^1^), fir (99.7 mg GAE 100 g^−1^), chestnut (52.86 mg GAE 100 g^−1^), arbutus (52.32 mg GAE 100 g^−1^) and manuka honey (88.71 mg GAE 100 g^−1^). Cotton and fir honey outreached the manuka honey in total phenols. Still, these two honeys (cotton and fir) showed high H_2_O_2_ levels superior to manuka honey. The total phenols and hydrogen peroxide showed synergistic antimicrobial activity. 

The phenolic compounds of honey are mainly acids and exert their antibacterial activity in different ways [69]. For example, coffeic acid acts through increased oxidative stress [70], chlorogenic acid increases the membrane permeability [71], while gallic acid causes cell membrane disruption and increased pore formation [72]. Flavonoids act in different ways as well, like, e.g., luteolin, which inhibits DNA helicases [73], and galangin, which inhibits peptidoglycan and ribosome synthesis [74].

The total flavonoids (TFC) (Table 1) were found higher in arbutus (4.22 mg CE 100 g^−^^1^), followed by fir honey (4.03 mg CE 100 g ^−1^). Those values are close to manuka honey (4.1 mg CE 100 g^−1^). Similarly, arbutus and fir showed high H_2_O_2_ levels equal to manuka honey.

Finally, the free radical scavenging activities (DPPH) (Table 1) were determined. Manuka honey (5.1 mg GA kg^−1^ E) overcame all other honeys, followed by fir (3.19 mg GAE kg^−1^), arbutus (2.78 mg GAE kg^−1^) and oregano (2.65 mg GAE kg^−1^) honey.

Assessing the above results, the degree to which bacterial growth is inhibited by honey was related to the coupling action of phenols and hydrogen peroxide.

Tyrosinase inhibition was evaluated, as well as the kojic acid levels (Table 2). As known, enzyme inhibition is applied successfully to multiple medicines for treating disease [75] through the targeting of a specific enzyme. Several foods showed an enzyme inhibitory activity. Tyrosinase inhibition impacts profitably upon hypertension, type 2 diabetes and obesity [76].

*Aspergillus flavus* is often isolated from worker bees and seems to produce a dominant secondary metabolite, which is kojic acid, almost identical to flufuran [77]. Moreover, kojic acid as a natural metabolite issued from fungi can inhibit tyrosinase activity in the synthesis of melanin [78] and is used in medical applications due to its antimicrobial and antiviral, antitumor, antidiabetic and anticancer activities [78]. In our study, manuka outperformed oregano honey, which showed high levels of tyrosinase inhibition and kojic acid. Tyrosinase is an enzyme that generates melaninogenesis and is involved in cancer and Parkinson’s disease [78] Yet, they found tyrosinase inhibition by structurally related flavonoids in tyrosinase [78]. It has been reported that flavonoids possessing several hydroxyl groups increase the tyrosinase inhibitory effects [78] due to the occurring interactions and chelation properties for copper ions by the hydroxyl group of flavonoids [79,80,81]. We assume that the inhibition of bacterial growth by honey can be a combination of tyrosinase inhibitory activity and flavonoids.

The antimicrobial action of honey is strongly influenced by geographical and seasonal issues [17]. Furthermore, the botanical origin and conditions of the harvesting, processing or storage of honey could be detrimental to its antibacterial actions [17].

Locally produced genuine honeys may have enhanced antibacterial activity and broad spectrums [82] and may be a valuable source for biomedical applications, therapeutic purposes for infections, as well as a functional food [83,84,85,86,87,88,89].

Sample treatments, different methodologies applied and variations of the properties of bacterial strains may be involved in the display of antimicrobial activity [75]. As stated, our multi-resistant strains come from bloodstream infections in hospitalized patients of a tertiary care university hospital. Samples treatment methodologies have different impacts upon the inhibition zones of the different strains [88,89,90,91]. Chloroform and n-hexane extracts seem to possess the most potent antibacterial activity [92]. 

Honey is not sterile [93]. Microorganisms are introduced in honey and its products either by botanical sources or honeybee’s microbiota or during processing techniques [1,93,94,95,96]. Bacteria in honey could produce secondary metabolites with antimicrobial activity [97]. Yet, when evaluating the antimicrobial activity of honey, a major issue must be considered. Unfortunately, several beekeepers use antibiotics for prophylactic or therapeutic purposes. Bacteria isolated from honey exhibited resistance profiles for commonly used antibiotics, such as vancomycin, ampicillin, oxacillin and ceftiofur, while a high prevalence of *S. aureus* subsp. *aureus* and *Bacteroides subtilis*-resistant strains were found [47]. Certainly, cutting edge technologies such as omics, RNA sequencing and metabolomics could permit a better investigation of the honeys. 16S rRNA gene sequencing could permit us to better investigate the honey microbiota and define its source of origin [21,98,99]. It is of note that 80.4% of the detected strains belonged to the genus *Bacillus* [95]. *Bacillus* species are facultatively anaerobic. They possess endospores that provide them with survival resistance in different environmental milieu. By the use of proteomics technology, honey glycoproteins with antibacterial effects were identified [98,99]. Yet, honey glycoproteins showed potent agglutinating and bactericidal activity by damaging the cell wall of the tested bacteria [100].

As shown in Figure 5a–d (Tables S9–S12 in the Supplementary Material), the different extracts caused different impacts on the MICs of the tested bacteria. The polarity of the solvents differs depending on their molecular structure, which determines their chemical behavior [101]. In every extraction, every solvent extracts a different mixture of chemical compounds [102]. This mixture obviously exerts a different antibacterial effect due to the different antibacterial potencies of each of their compounds. Some compounds of a honey with an enhanced antibacterial effect [103,104] may not be extracted by a different solvent or they may be extracted in smaller quantities, and thus, the antibacterial effect of two or more extracts of the same honey but by different solvents could differ [102]. Furthermore, some compounds may be common to all extracts but in different quantities, so perhaps there are also dose–response issues [102]. These remarks are supported by the results shown in the aforementioned tables. In Table S11, we show that the n-hexane chestnut extract has a higher MIC value than the n-hexane arbutus extract, while their ethyl acetate extracts have identical MIC values.

In this study, we focused our research on clinical multi-resistant Gram-negative bacteria due to their medical importance, together with the fact that less research has been oriented in this direction [37]. 

Our results showed inhibitory activity against multidrug-resistant Gram-negative bacteria. The antimicrobial activity was closely related to the type of honey, its dilution and treatment methodology and the microorganism [105]. Arbutus honey showed higher antimicrobial activity against *E. cloacae* subsp. *dissolvens* in the 75% concentration, while fir honey was more lethal for this same bacterium in the 25% concentration. It is of note that several Greek honeys in our study outperformed manuka honey. It is remarkable that, for any given type of honey and for any given concentration in the well diffusion method, no significant differences were identified (one-way ANOVA for *p* < 0.005) among all four multidrug-resistant Gram microorganisms, which explains that the destructive effect on bacterial cells was the same regardless of the species or bacterial strain. Overall, honey deploys its antimicrobial activity regardless of the microorganism, suggesting a non-specific action. On the other side, as stated in our previous work [20], there are important chemical compositional differences in the honey types due to the various botanical and topographical sources [106,107]. 

Our research sheds light on the antimicrobial potential of several rare variations of raw honey that could eventually function as antibiotic adjuvants to treat infections. 

## 4. Materials and Methods

**Figure 7:**
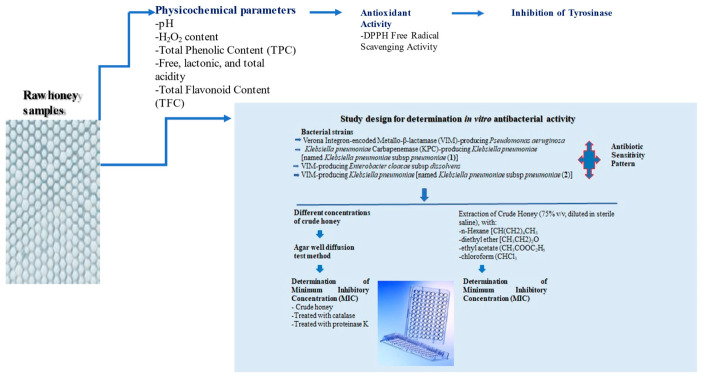
Schematic analysis of the workflow.

The overall workflow for the samples collection and analyses performed is presented in Figure 7.

### 4.1. Study Area—Honey Samples

Raw honey samples (*n* = 47) of different botanical origin were collected from local beekeepers from different geographical areas in Greece: Epirus, Evros, Thessalia and Attiki Provinces. The botanical source and geographical location of the different types of honey are listed in Table 6. Honeys are classified according to the plant species that dominate their geographical origin during the harvest season following information from the beekeepers who provided us with honey. Beekeepers tend to carry the hives during the flowering period each year to places where these species of plants predominate in mountainous fields. They provided us with 300 g of genuine honey samples, which was collected in a sterile container and kept at 2–8 °C in a dark place to prevent photodegradation until experimentation. In the present study, honeys were estimated for their microbiological quality by dissolution in cation-adjusted Mueller–Hinton broth (CAMHB; Oxoid, Ltd., Basingstoke, Hampshire, England) and then inoculated into sheep blood agar (Columbia agar base with 5% sheep blood, Becton Dickinson) and incubated aerobically at 37 °C for 48 h. Samples showing bacterial or yeast growth were not eligible for this study. Thus, 5 samples were excluded from our study, leaving 42 eligible samples.

#### 4.1.1. Control Indexes of the Experimental Design

Manuka honey MGO 550+ (Manuka Health, Auckland, New Zealand) was provided for our study as a positive control. Yet, we prepared artificial honey based on the predominant sugars in honey [77,78,79] as follows: 3 g of sucrose, 15 g of maltose, 81 g of D-fructose and 67 g of D-glucose (Sigma-Aldrich, St. Louis, MO, USA) in 34 mL of sterile water.

#### 4.1.2. Determination of Physicochemical Parameters

##### Determination of pH

An aliquot of 10 g of each honey was diluted in 75 mL of CO_2_-free distilled water. The pH was measured by the aid of a portable pH meter (Hanna instruments, HI98100 checker plus) [26,108,109,110]. The pH meter was calibrated with two standard identification buffers prior to analysis: pH 4 and pH 10, following the manufacturer’s instructions. Each measurement was performed in triplicate.

##### Determination of H_2_O_2_ Content

The H_2_O_2_ content was determined using Megazyme GOX assay kit microplates (Megazyme International Ireland Ltd., Bray, Co. Wicklow, Ireland). All honey samples were tested in triplicate. Determination of the H_2_O_2_ content is based on the release of H_2_O_2_. after glucose oxidase catalysis of the oxidation of β-D-glucose to D-glucono-δ-lactone [101,102]. Studies reported that highest accumulation of H_2_O_2_ in honey solutions was shown for honeys diluted to 30 and 50% [83,84]. Therefore, we prepared 40% (*w*/*w*) honey solutions diluted in 0.1-M potassium phosphate buffer (pH 7.0). The absorbance of the microplate wells was measured at 510 nm on the reader [109,110]. H_2_O_2_ concentration was calculated by the aid of a standard curve from the 200-μM H_2_O_2_ stock solution. Yet, alongside the serial dilutions of honey a standard curve was run. All honey and standard curve samples were run in triplicate.

##### Determination of the Total Phenolic Content (TPC) (Measurement of Level of Phenolic Compounds, Which Contribute to the Antibacterial Activity of Honey)

Folin–Ciocalteu method as modified [111,112] was applied for measuring of TPC. Initially, 20 µL volume of each sample was diluted in 1 mL of ultrapure water and then 100 µL of Folin–Ciocalteu reagent (Merck KGaA, Darmstadt, Germany) was added. After 3 min, 280 µL of 25% *w*/*v* sodium carbonate solution (280 µL) together with 600 µL of ultrapure water (1.7 mL) were added to the mix. Optical density was measured at 765 nm against a blank containing Folin–Ciocalteu reagent and ultrapure water after 24 h incubation in the dark at room temperature. A standard gallic acid curve (50–1500 µg/mL) was prepared for determination of the total phenolic content (TPC). Results are expressed as gallic acid equivalents (GAEs) by the aid of standard gallic acid curve versus TPC concentration in mg GAE/100 g of honey.

##### Determination of Free, Lactonic and Total Acidity (Determination of the Acidity Factors with May Have a Potential Effect on the Expression of the Antimicrobial Activity of Honey)

The free, lactonic and total acidity were specified by the titrimetric method according to AOAC Official Methods [113] and results are expressed to meq/kg.

##### Determination of the Total Flavonoid Content (TFC) (Due to the Contribution of Flavonoids in Antimicrobial Activity of Honey)

The aluminum chloride method was applied to determine the TFC [114,115]. Initially, honey solution (1 mg/mL) was mixed with 0.3 mL NaNO_2_ (5%) followed by addition of a solution of 0.3 mL AlCl_3_ (10%) after 5 min. At the end, all honey samples were neutralized with a 2 mL of NaOH solution (1 M). The absorbance of the samples was measured at 510 nm by the aid of a spectrophotometer (Quercetin; Sigma–Aldrich, St. Louis, MO, USA). A standard quercetin curve (20–100 mg/L) was prepared for determination of the Total Flavonoid Content (TFC). Results are expressed as Quercetin Equivalents (QE)/100 g of honey [116]. Each measurement was performed in triplicate.

##### Determination of the DPPH Free Radical Scavenging Activity (Screening the Antioxidant Activity of Honey Samples through Investigation of the Overall Hydrogen or Electron Donating Activity of Single Antioxidants)

To determine the DPPH radical scavenging activity, 1 g of each honey was dissolved in 5 mL of methanol 40% (*v*/*v*, with acidified water) and mixed in a magnetic stirrer for 15 min to evaluate antioxidant activity [117] by assay of 1,1-diphenyl-2-picrylhydrazyl (DPPH). Therefore, 35 µL of honey mixture was added with 250 µL of DPPH solution (2 mg DPPH (Sigma–Aldrich, St. Louis, MO, USA)/100 mL) and left in the dark at 25 °C for 30 min. The absorbance of the samples was measured at 517 nm by means of a spectrophotometer, against blank methanol and ascorbic acid using as a positive control. A standard ascorbic acid curve (0–10 mg/L). was created to determine the DPPH Free Radical Scavenging Activity, as the concentration of honey sample required to scavenge 50% of DPPH (EC50). Each measurement was performed in triplicate. DPPH scavenging activity (%) was determined using the following equation:

$$\mathrm{DPPH}\;\mathrm{scavenging}\;\mathrm{activity}\;\left(\%\right)=\frac{\mathrm{Acontrol}\;-\;\mathrm{Asample}}{\mathrm{Acontrol}}\times 100$$

##### Determination of the Anti-Tyrosinase Activity (Study of Tyrosinase inhibition by Honey Samples, Factor That Potentially Enhances the Antimicrobial Activity)

To determine the anti-tyrosinase activity, the honey was diluted in 20% ethanol to obtain a final concentration of 50% [118,119]. Then, 50 μL of the above dilution was added 150 μL of 0.02-M phosphate buffer (pH 6.8) and 50 μL of mushroom tyrosinase (TYR) (313 Units/mL in phosphate buffer, Sigma-Aldrich, St. Louis, MO, USA) and incubated at 37 °C for 10 min. Afterwards, 50 μL of 3,4-Dihydroxy-Lphehylalanine (0.32 mM) (L-Dopa, Sigma Sigma–Aldrich, USA) was added to the wells and anti-tyrosinase activity was specified at 492 nm following incubation at 25 °C for 2 min. Kojic acid (Merck KGaA, Darmstadt, Germany) was applied as the standard inhibitor of the enzyme tyrosinase. The inhibition activity (%) was determined using the following equation:

$$\%\;\mathrm{Inhibition}\;=\frac{\left(A-B\right)-\left(C-D\right)}{A-B}\times 100$$

where: A was the Optical Density (OD492) of the control (L-Dopa mixed with tyrosinase enzyme in buffer), B; represented the blank (L-Dopa in buffer), C; represented the reaction of L-Dopa with tyrosinase enzyme and honey in buffer; and D represented the blank of C (L-Dopa mixed with honey in buffer).

### 4.2. Study Design for Determination In Vitro Antibacterial Activity of Honey Samples

#### 4.2.1. Bacterial Strains and Antibiotic Sensitivity Pattern 

Four genotypically confirmed carbapenemase positive clinical strains were included in this study as follows (Figure 6): (1) VIM-producing *P. aeruginosa*, (2) KPC-producing *K. pneumoniae* (named *Klebsiella pneumoniae* subsp. *pneumoniae* (1), (3) VIM-producing *E. cloacae* subsp. *dissolvens* and (4) VIM-producing *K. pneumoniae* (named *Klebsiella pneumoniae* subsp. *pneumoniae* (2). The strains were provided by the Bacterial Collection of the Department of Microbiology, Medical School, National and Kapodistrian University of Athens, isolated from bloodstream infections of separated patients hospitalized in tertiary care hospitals located in the broad Attica region. The identification of all isolates was confirmed by using the Matrix-assisted laser desorption ionization time of flight mass spectrometry (MALDI-TOF) on a Microflex LT (Bruker Daltonics, Bremen, Germany) platform. Antimicrobial susceptibility testing and minimum inhibitory concentration (MIC) values were determined by VITEK 2 and Etest (bioMérieux, Marcy l’Etoile, France). The presence of *bla*_KPC_ and *bla*_VIM_ was determined using previously described oligonucleotide primers and cycling conditions [120,121].

#### 4.2.2. Used Solvents

The following solvents were used in our study to the order of increasing polarity; n-Hexane [CH(CH2)_4_CH_3_; for analysis EMSURE^®^ ACS, Merck, Germany], diethyl ether [CH_3_CH2)_2_O; for analysis EMSURE^®^ ACS, Merck, Germany], ethyl acetate (CH_3_COOC_2_H_5_; for analysis EMSURE^®^ACS, ISO, Reag. Ph Eur, Merck, Germany), chloroform (CHCl_3_; for analysis EMSURE^®^ACS, ISO, Reag. Ph Eur, Merck, Germany) and distilled water.

#### 4.2.3. Extraction of Crude Honey

The Separation Funnel Method was to determine the antibacterial activity of the different types of extraction of raw honey samples based on polarity. Initially, 100 g of crude honey was diluted in 150 of sterile distilled water, placed in a Stomacher sterile bag and mixed. Transfer to a 500-mL separatory funnel, shake and allow to precipitate. 50 mL of the less polar solvent n-hexane was added and shaken for 15 min and reprecipitated to allow the solvent layers to separate. Through the bottom of the funnel, the aqueous layer was removed, and the top layer was collected to obtain a n-hexane fraction. Three successive extracts were performed by applying an equal volume of n-hexane followed by shaking and separation. In the same manner, diethyl ether, ethyl acetate and chloroform were extracted to obtain diethyl ether, ethyl acetate and chloroform fractions. All 3 successive layers were thoroughly mixed and the water contaminating extracts were ward off by filtration over anhydrous sodium sulfate. The last step involves collecting the organic solvent extract and concentrating it by evaporation under reduced pressure using a rotary evaporator (KNF RC 900, KNF Neuberger GmbH, Breisgau, Germany) at 40 °C, 30 °C, 60 °C and 50 °C for n-hexane, diethyl ether, ethyl acetate and chloroform, respectively [116,117].

#### 4.2.4. Determination of the Antibacterial Activity of Crude Honey Samples

The samples were diluted in sterile saline at different concentrations. 75%, 50%, 25%, 12.5% and 6% following the agar well diffusion test method [48,122,123,124]. In this vein, bacterial cell suspensions were prepared from overnight cultures at 37 °C on brain–heart infusion agar (BHI; Oxoid, Ltd., Basingstoke, Hampshire, England). Wells (6.2 mm diameter) were immerged into the agar to place 50 µL of the honey sample followed by anaerobic conditions at 37 °C for 72 h. The diameter of the inhibition zones created around the wells showed the antibacterial activity and was measured (mm). Honeys showing an inhibition zone over 8mm were diluted twice (1:2 and up to 1:8) to reach the endpoint. The negative control was prepared with sterile dH_2_O solution. Each experiment was performed in triplicate.

##### Determination of Minimum Inhibitory Concentration (MIC)

The minimum inhibitory concentration (MIC) of the honey types was determined in sterile 96-well polystyrene microtiter plates (Kisker Biotech GmbH and Co. KG, Steinfurt, Germany) by using a spectrophotometric bioassay, as previously described [87,124]. Briefly, overnight bacterial cultures grown in Mueller–Hinton broth (MHB; Oxoid, Ltd., Basingstoke, Hampshire, England). were adjusted to a 0.5 McFarland turbidity standard (~1.5 × 10^8^ CFU/mL). Approximately 5 × 10^4^ CFUs in 10 µL Mueller–Hinton broth were added to 190 µL of 2-fold diluted test honey (honey concentration ranged from 75 to 0.58% *w*/*v*) in Mueller–Hinton broth. Two-fold serial dilutions of the same range of manuka honey and artificial honey were included for comparison. The control wells contained only Mueller–Hinton broth-inoculated with bacteria. The optical density (OD) was determined at 630 nm using a microplate reader (Multi-detection reader, BioTek^®^, Winooski, VT, USA), just prior to incubation (t = 0) and after 24 h of incubation (t = 24) at 37 °C. MIC was determined as the lowest honey concentration that results in 100% growth inhibition.

##### Determination of Minimum Inhibitory Concentration (MIC) Performed after Enzymatic Treatment of Honey Samples with Catalase and Proteinase K

This determination was performed in two ways:

(a) Honey samples were treated with catalase which degrades hydrogen peroxide, allowing evaluation of the contribution of hydrogen peroxide production to antibacterial activity [110].
(b) The addition of proteinase K permits assessment of antimicrobial activity due to proteins and peptides [109].

The catalase solution was set by dilution of catalase powder (30 mg) from bovine liver (SERNA, Heidelberg, Germany) to 10 mL phosphate buffer (pH 7.4. In 1.5 mL honey 50% *v/v* (750 µL honey and 750 µL Muller Hinton Broth), 28 µL of the stock solution were added to obtain a final concentration of 600 U/mL. Diluted honey was shacked in an incubator for 16 h at 37 °C in 210 rounds. The proteinase K stock solution was set by dilution of proteinase powder (10mg) (Ambion^®^, Inc., Huntingdon, Cambridgeshire, UK) in 1-mL distilled water to obtain a final concentration of 10 mg/mL. Then, all treated honeys solutions by catalase and proteinase K were determined the MIC values, as reported previously in the section “Determination of Minimum Inhibitory Concentration (MIC)”. Controls were prepared; no honey control as a positive control and no honey control with catalase or proteinase K (catalase control/proteinase K control) in order to evaluate their effect upon the bacterial growth. MIC values of the treated honeys when compared to the untreated honey reveal the presence of hydrogen peroxide and/or proteinaceous aggregates, which redound to the antibacterial activity of honeys. All experiments were done in triplicate.

#### 4.2.5. Screening the Antibacterial Efficacy of Honey Extracts Antimicrobial Assay

##### Preliminary Assessment of Antimicrobial Activity 

The antibacterial efficacy of honey extracts from n-hexane, diethyl ether, chloroform and ethyl acetate were evaluated in vitro using the agar well diffusion assay. Honey samples proved to be most active at 75% *v*/*v* and, thus, tested against multi-resistant bacterial strains. Negative controls (pure solvent; n-hexane, diethyl ether, chloroform and ethyl acetate) were performed equally, as well as the solvent control, which showed no activity. The diameter of the inhibition zones was measured (mm).

##### Minimum Inhibitory Concentration (MIC)

All 4 crude honey extracts were determined their minimum inhibitory concentration (MIC) by microtiter method using a spectrophotometer (Kisker Biotech GmbH and Co. KG, Steinfurt, Germany) [121]. The wells were immersed in BHI broth (BHI; Oxoid, Ltd., Basingstoke, Hampshire, UK), where two-fold dilutions of honey extract (0.001–12% *v*/*v*) were placed. Multi-resistant bacterial strains were tenfold diluted with sterile saline solution and 20 µL of a bacterial suspension (1 × 10^8^ colony-forming units (CFUs)/mL, 0.5 McFarland standards) was placed in each well and then sealed and incubated at 37 °C for 72 h. In the same manner, they were prepared for comparisons two-folds Manuka and artificial honey. Mueller-Hinton was poured into control wells and inoculated with bacteria.

By the aid of a microplate reader (Multi-detection reader, BioTek^®^), the optical density (OD) was measured at 630 nm before and after 24 h incubation at 37 °C. Finally, the MIC was recognized as the lowest concentration of honey resulting in 100% growth inhibition.

- Points needs clarification

(i) Crude honey: Honey sample (10 g) was extracted in a condenser with 50 mL of distilled water at 60 °C for more than 6 h. The obtained extract was filtered to remove particles and volume was adjusted with ultra-pure water.
(ii) Dried honey: After extraction with diethyl ether, dried honey was applied in the MIC assay as following; weights extracts were diluted (400 mg/mL honey/ultrapure water solution) for 24 h, vortexed at 1500 rpm for 3 min and filtered through a 0.45 Whatman TM syringe filter (Merck, Germany).Then, serial dilutions were prepared at the following concentrations (200 mg/mL, 100 mg/mL, 50 mg/mL, 25 mg/mL, 12.5 mg/mL, 6.25 mg/mL, 3.125 mg/mL, 1.56 mg/mL, 0.78 mg/mL and 0.39 mg/mL).

##### Statistical Analysis

Analysis of variance with one way ANOVA and Tukey’s HSD post hoc comparison was used to compare more than two groups of samples, while in the case of two groups of samples, the Mann–Whitney (Wilcoxon) nonparametric test was applied. Spearman Rho correlation coefficient was estimated when the degree of correlation was required. All analyses were performed with Microsoft Excel at an alpha of 95%.

## 5. Conclusions

Although honey is known to have multiple successful uses in medicine, food and cosmetics, there is still missing information on the mechanisms of antimicrobial action, as it seems to be a multifactorial process. While some authors have reported the effects of honey directly upon the bacterial membrane by impacting the membrane integrity and metabolic activity, others have reported the potential role of the enzymatic production of hydrogen peroxide, its acidic pH, the phenolic content, its minerals, the levels of MGO, some biodrastic proteins and, eventually, certain beneficial microorganisms secreting antimicrobial substances in combat bacteria. Our results showed that there are significant variations in the physical and chemical characteristics of the various types of honey, depending on their floral origin. Furthermore, the different honeys showed important differentiations concerning their antibacterial effects, as demonstrated by the well diffusion assay, and many of them outperformed the antimicrobial potency of manuka honey. The addition of catalase caused significant differences among the types of honeys in their antibacterial activity, assessed by the MIC method, implying that hydrogen peroxide is involved in the pathways that lead to the formation of compounds with antibacterial properties. The extraction process of the honeys by four organic solvents demonstrated that each solvent extracted different mixtures of compounds, and hence, the antibacterial activities of the extracts were different. This finding implies that there is more than one responsible substance and that the final antibacterial effect is probably dose-responsive. 

## Figures and Tables

**Table 1:** Physical and chemical characteristics of the different honeys (values in columns with the same superscript letters are significantly different) by one-way ANOVA and Tukey’s HSD test with a significance level of *p* < 0.05.

Honey Samples Based on Botanical Origin	Physicochemical Parameters						
	pH	H_2_O_2_ mM in 40% Aqueous Honey Solution	Free Acidity	Lactonic Acidity	TPC (mg GAE/100 g of Honey)	TFC mg Quercetin Equivalents (CE)/100 g of Honey	DPPH (mg GAE/kg of Honey)
Cotton	3.73 ± 0.33 ^a^	0.84 ± 0.12 ^a^	17.75 ± 2.48 ^a^	8.30 ± 4.07	99.7 ± 39.89 ^a^	3.33 ± 0.93 ^a^	1.9 ± 1.22 ^a^
Arbutus	3.32 ± 0.22 ^b^	0.97 ± 0.15 ^b^	42.24 ± 6.25 ^a,b^	11.88 ± 4.08	52.32 ± 19.21 ^b^	4.22 ± 0.64 ^b^	2.78 ± 0.66 ^b^
Chestnut	3.66 ± 0.49 ^c^	1.16 ± 0.06 ^c^	42.80 ± 14.01 ^a,c^	15.44 ± 4.18	52.86 ± 23.91 ^c^	3.28 ± 1.95 ^c^	2.02 ± 1.33 ^c^
Thyme	3.48 ± 0.42 ^d^	0.59 ± 0.08 ^d^	37.60 ± 11.97 ^a,d^	11.14 ± 1.97	70.7 ± 25.18	2.06 ± 0.80 ^b,d^	1.5 ± 0.69 ^d^
Orange	3.70 ± 0.18 ^e^	0.36 ± 0.06 ^e^	30.48 ± 5.19 ^e^	9.23 ± 0.66	38.3 ± 7.16 ^a,d^	1.1 ± 0.18 ^a, b, c, e^	0.9 ± 0.25 ^b,e^
Oregano	3.23 ± 0.26 ^f^	1.39 ± 0.31 ^f^	42.18 ± 7.26 ^a, f^	12.45 ± 1.50	46.00 ± 14.80 ^a,e^	3.58 ± 0.77 ^e,f^	2.65 ± 0.94 ^e,f^
Fir	4.87 ± 0.21 ^a,b,c,d,e,f,g,j^	3.02 ± 1.78 ^a,b,c,d.e,f,g,j,k^	18.39 ± 4.66 ^b,c,d,f,g^	11.04 ± 3.54	99.1 ± 34.43 ^b,c,d,e,f^	4.03 ± 0.73 ^d,e,g^	3.19 ± 0.60 ^e,g^
Sunflower	3.93 ± 0.25 ^b,h,f^	0.36 ± 0.05 ^g^	36.93 ± 3.69 ^a,g,j^	9.70 ± 0.51	30.6 ± 1.71 ^a, f,g^	0.92 ± 0.14 ^a,b,c,f,g,h^	1.33 ± 0.48 ^d,g,h^
Heath	3.22 ± 0.13 ^j^	1.29 ± 0.11 ^j^	15.28 ± 2.52 ^b,c,d, e,f,j^	7.84 ± 2.14	43.66 ± 9.87 ^a,f^	1.36 ± 0.31 ^a,b,c,f,g,j^	1.98 ± 0.99 ^j^
Manuka honey	4.10 ± 0.15 ^b,d,f,j^	1.27 ± 0.74 ^k^	15.2 ± 0.20 ^b,c,d,e,f,j^	7.10 ± 0.20	88.71 ± 0.3 ^d,g^	4.1 ± 0.80 ^d,e,h,j^	5.1 ± 0.8 ^a,b,c,d, e,f,g,h,j^

**Table 2:** Determination of tyrosinase inhibition activity and kojic acid from different types of honey (values in columns with the same superscript letters are significantly different) by one-way ANOVA and Tukey’s HSD test with a significance level of *p* < 0.05.

Honey Samples Based on Botanical Origin	Tyrosinase Inhibition	Kojic Acid
Cotton	56.21 ± 3.02 ^a^	31.24 ± 1.91 ^a^
Arbutus	48.66 ± 9.08 ^b^	18.74 ± 4.56 ^b^
Chestnut	58.28 ± 4.61 ^c^	40.28 ± 2.64 ^b,c^
Thyme	50.04 ± 1.87 ^d^	14.31 ± 0.76 ^a,c,d^
Orange	44.39 ± 2.90 ^c,e^	7.84 ± 1.93 ^a,c,e^
Oregano	81.92 ± 4.90 ^a,b,c,d,e,f^	86.21 ± 14.48 ^a,b,c,e,f^
Fir	56.46 ± 12.56 ^f,g^	19.66 ± 9.42 ^c,f,g^
Sunflower	29.40 ± 2.85 ^a,b,c,d,e,f,g,h^	4.99 ± 1.12 ^a,b,c,f,g,h^
Heath	40.09 ± 2.96 ^a,c,f,g,j^	7.32 ± 2.54 ^a,c,f,g,j^
Manuka honey	85.11 ± 4.33 ^a,b,c,d,e,g,h,j^	210.15 ± 12.26 ^a,b,c,d,e,f,g,h,j^

**Table 3:** Correlation between the physicochemical parameters of the honeys (Spearman’s rho coefficient, statistical significance for *p* < 0.05).

Physicochemical Parameters	Indexes of Correlation								
pH	-	-	-	-	-	-	-	-	-
H_2_O_2_	-	-							
Free acidity	-	-	-						
Lactonic acidity	-	-	r = 0.952 *p* < 0.00001	-					
TPC	-	-	-	-	-				
TFC	-	-	-	-	-	-			
DPPH	-	r = 0.802 *p* = 0.00521	-	-	-	r = 0.89 *p* = 0.00054	-		
Tyrosinase inhibition	-	-	-	-	r = 0.636, *p* = 0.04791	r = 0.872, *p* = 0.03304	r = 0.696, *p* = 0.0251	-	
Kojic acid	-	-	-	-	-	-	r = 0696, *p* = 0.0251	r = 0.975, *p* < 0.0001	-
	pH	H_2_O_2_	Free acidity	Lactonic acidity	TPC	TFC	DPPH	Tyrosinase inhibition	Kojic acid

**Table 4:** Samples of the different honey types outperforming manuka honey’s antibacterial activity in the well diffusion assay (chi-square, statistical significance level *p* < 0.05).

Bacterial Species	Number of Samples in Well Diffusion Assay					*p*-Value
	Concentration of Honey (% *v*/*v*)					
	75%	50%	25%	12.5%	6.25%	
*Enterobacter cloacae* subsp. *dissolvens*	2	8	16	18	5	<0.001
*Pseudomonas aeruginosa*	7	6	23	9	9	<0.001
*Klebsiella pneumoniae* subsp. *pneumoniae* (1) *	6	11	30	10	-	<0.001
*Klebsiella pneumoniae* subsp. *pneumoniae* (2) **	5	19	31	12	2	<0.001

* KPC-producing *K. pneumoniae* (named *Klebsiella pneumoniae* subsp. *pneumoniae* (1); ** VIM-producing *K. pneumoniae* (named *Klebsiella pneumoniae* subsp. *pneumoniae* (2).

**Table 5:** Samples of the different honey types outperforming manuka honey’s antibacterial activity in the MIC assessment method (chi-square, statistical significance level *p* < 0.05).

Bacterial Species	Number of Samples in the MIC_95_ Values (% *v*/*v*) Assessment			*p*-Value
	Crude Samples	Catalase Addition	Protease Addition	
*Enterobacter cloacae* subsp. *dissolvens*	8	41	19	<0.0001
*Pseudomonas aeruginosa*	5	2	1	*p* = 0.178575
*Klebsiella pneumoniae* subsp. *pneumoniae* (1) *	1	31	18	<0.0001
*Klebsiella pneumoniae* subsp. *pneumoniae* (2) **	6	29	17	<0.0001

* KPC-producing *K. pneumoniae* (named *Klebsiella pneumoniae* subsp. *pneumoniae* (1); ** VIM-producing *K. pneumoniae* (named *Klebsiella pneumoniae* subsp. *pneumoniae* (2).

**Table 6:** Botanical source and geographical location of the honey types.

Honey Number	Botanical Source	Geographical Location	Honey Number	Botanical Source	Geographical Location
1	Cotton	Karditsa	23	Orange	Evros
2	Cotton	Evros	24	Oregano	Epirus
3	Cotton	Epirus	25	Oregano	Epirus
4	Cotton	Epirus	26	Oregano	Epirus
5	Arbutus	Arkadia	27	Oregano	Epirus
6	Arbutus	Epirus	28	Fir	Ftiotida
7	Arbutus	Epirus	29	Fir	Epirus
8	Arbutus	Evros	30	Fir	Epirus
9	Arbutus	Evros	31	Fir	Epirus
10	Chestnut	Epirus	32	Fir	Epirus
11	Chestnut	Epirus	33	Fir	Epirus
12	Chestnut	Epirus	34	Fir	Epirus
13	Chestnut	Evros	35	Sunflower	Evros
14	Chestnut	Evros	36	Sunflower	Evros
15	Thyme	Epirus	37	Cotton & Sunflower	Evros
16	Thyme	Attica (Laurion)	38	Heath	Epirus
17	Thyme	Epirus	39	Heath	Epirus
18	Thyme	Epirus	40	Fir & Heath	Arkadia
19	Thyme	Epirus	41	Heath	Epirus
20	Orange	Epirus	42	Heath	Epirus
21	Orange	Epirus	43	AM HEALTH Manuka Health MGO™550+ (25+)	Lower Hutt, New Zealand
22	Orange	Epirus			

## Data Availability

Not applicable.

## Supplementary Materials

The following supporting information can be downloaded at https://www.mdpi.com/article/10.3390/antibiotics11030422/s1: Table S1. Antibacterial activity (zone of inhibition in mm) of various concentrations of the different honeys against *Enterobacter cloacae* subsp. *dissolvens* assessed by the well diffusion method. Table S2. Geometrical means of the antibacterial activity of the different honeys against *Enterobacter cloacae* subsp. *dissolvens* assessed by the MIC method as the crude sample, with the addition of catalase and proteinase K. Table S3. Antibacterial activity (zone of inhibition in mm) of various concentrations of the different honeys against *P. aeruginosa* assessed by the well diffusion method. Table S4. Geometrical means of the antibacterial activity of the different honeys against *P. aeruginosa* assessed by the MIC method as the the crude sample, with the addition of catalase and proteinase K. Table S5. Antibacterial activity (zone of inhibition in mm) of various concentrations of the different honeys against *Klebsiella pneumoniae* subsp. *pneumoniae* (1) assessed by the well diffusion method. Table S6. Geometrical means of the antibacterial activity of the different honeys against *Klebsiella pneumoniae* subsp. *pneumoniae* (1) assessed by the MIC method as the crude sample, with the addition of catalase and proteinase K. Table S7. Antibacterial activity (zone of inhibition in mm) of various concentrations of the different honeys against *Klebsiella pneumoniae* subsp. *pneumoniae* (2) assessed by the well diffusion method. Table S8. Geometrical means of the antibacterial activity of the different honeys against Klebsiella pneumoniae subsp. pneumoniae (2) assessed by the MIC method as the crude sample, with the addition of catalase and proteinase K. Table S9. Geometrical means of the MIC values of the honey extracts by different solvents against *P. aeruginosa.* Table S10. Geometrical means of the MIC values of the honey extracts by different solvents against *K. pneumoniae* subsp. *pneumoniae* (1). Table S11. Geometrical means of the MICs of the honey extracts by different solvents against *E. cloacae* subsp. *dissolvens.* Table S12. Geometrical means of the MIC values of the honey extracts by different solvents against *K. pneumoniae* subsp. *pneumoniae* (2).
