# Supplementary material for: Antimicrobial Evaluation of Various Honey Types against Carbapenemase-Producing Gram-Negative Clinical Isolates
Source: Antibiotics (Basel). 2022 Mar 21;11(3):422. doi: 10.3390/antibiotics11030422 (PMC8944737; doi:10.3390/antibiotics11030422)
Supplement: Supplementary file 1 [file antibiotics-11-00422-s001.zip › antibiotics-1618395-supplementary.pdf]

Supplementary Materials

Table S1. Antibacterial activity (zone of inhibition in mm) of various concentrations of the different honeys against *Enterobacter cloacae* subsp *dissolvens* assessed by the well diffusion method.

Honey type	**Inhibition zone (in mm)**				
	**75%**	**50%**	**25%**	**12.5%**	**6.25%**
Cotton	16.15±3.55	13.95±2.51	11.7±1.48	10.03±1.5 (n=2)	9.4 (n=1)
Arbutus	18.46±4.19	15.26±2.41	12.28±2.11	11.00±1.62 (n=3)	9.2 (n=1)
Chestnut	15.44±1.63	13.32±1.80	11.48±1.19	9.25±0.21 (n=2)	0
Thyme	15.19±1.09	12.14±1.76	7.80±4.45	7.7 (n=1)	0
Orange	16.65±0.36	12.35±0.76	10.01±0.25	0	0
Oregano	14.38±0.61	13.15±1.60	11.53±1.70	9.8±1.27 (n=2)	0
Fir	15.79±1.28	14.17±1.00	12.44±1.24	10.46±1.67 (n=5)	9.05±0.15 (n=2)
Sunflower	13.67±0.39	11.90±0.31	8.75±3.94	0	0
Heath	15.88±2.84	13.12±2.44	10.16±2.72	9.7±2.12 (n=2)	8.7 (n=1)
Manuka	21.3	15.2	11.3	0	0
Artificial honey	6.2	0	0	0	0
Significance (0ne way ANOVA)	F=6.321064 P<0.0001	F=2.571789 P<0.05	F=3.343586 P<0.05	-	-

Table S2. Geometrical means of the antibacterial activity of the different honeys against *Enterobacter cloacae* subsp *dissolvens* assessed by the MIC method as crude sample, with the addition of catalase and protease.

**Honey type**	**MIC_{95} values (% v/v)**		
	**Crude samples**	**Catalase addition**	**Protease addition**
**Cotton**	5.26	8.84	7.43
**Arbutus**	4.74	6.25	6.25
**Chestnut**	4.74	8.25	8.25
**Thyme**	6.25	9.47	10.88
**Orange**	12.5	21.02	14.87
**Oregano**	7.43	8.84	8.84
**Fir**	5.13	6.90	6.90
**sunflower**	9.92	19.84	15.75
**Heath**	6.25	12.16	10.88
**Manuka**	6.25	25	12.50
**Artificial honey**	-	-	-
	-	F=12.035046 P<0.0001	-

Table S3. Antibacterial activity (zone of inhibition in mm) of various concentrations of the different honeys against *P. aeruginosa* assessed by the well diffusion method.

Honey type	**Inhibition zone (in mm)**				
	**75%**	**50%**	**25%**	**12.5%**	**6.25%**
Cotton	16.58±1.21	13.1±1.72	8.28±4.86 (n=4)	0	0
Arbutus	16.44±3.93	12.88±2.64	11.16±2.05	10.13±0.4 (n=3)	0
Chestnut	15.04±3.09	13.46±1.59	10.64±2.06	9.8±0.42 (n=2)	0
Thyme	15.72±3.75	13.40±1.75	11.92±1.93	13.4 (n=1)	0
Orange	13.38±0.52	11.85±0.43	5.3±5.3 (n=3)	0	0

Oregano	13.05±0.98	11.33±1.29	2.8±4.8 (n=2)	0	0
Fir	14.66±2.21	13.01±1.27	8.37±5.88 (n=5)	11.8 (n=1)	0
sunflower	13.23±0.73	11.73±0.74	3.23±3.96 ((n=3)	0	0
Heath	15.30±2.56	12.92±2.42	11.66±1.98	10.55±1.2 (n=2)	0
Manuka	18.2	14.4	14.4	0	0
Artificial honey	6.2	0	0	0	0
Significance (0ne way ANOVA)	F=2.590381 P=0.017902	-	F=4.058618 P=0.000805	-	-

Table S4. Geometrical means of the antibacterial activity of the different honeys against *P. aeruginosa* assessed by the MIC method as crude sample, with the addition of catalase and protease.

Honey type	MIC95 values (% v/v)		
	Crude samples	Catalase addition	Protease addition
Cotton	6.25	7.43	6.25
Arbutus	7.18	10.88	9.47
Chestnut	6.25	12.5	9.47
Thyme	6.25	8.25	8.84
Orange	8.84	42.04	12.5
Oregano	8.84	10.51	9.92
Fir	6.25	7.62	9.29
Sunflower	9.92	9.92	9.92
Heath	7.18	7.18	9.47
Manuka	6.25	6.25	6.25
Artificial honey	-	-	-
	-	F=16.369898 P<0.001	-

Table S5. Antibacterial activity (zone of inhibition in mm) of various concentrations of the different honeys against *Klebsiella pneumoniae* subsp *pneumoniae* (1) assessed by the well diffusion metho.

Honey type	Inhibition zone (in mm)				
	75%	50%	25%	12.5%	6.25%
Cotton	15.15±2.88	12.58±0.70	10.63±1.03	0	0
Arbutus	14.96±3.22	12.90±2.82	9.14±5.47	10.60±1.56 (n=2)	0
Chestnut	14.44±2.78	13.28±2.52	11.66±1.82	10.75±1.91 (n=2)	0
Thyme	13.88±0.76	12.46±0.54	10.24±1.33	9.4 (n=1)	0
Orange	13.63±0.90	11.80±1.22	4.98±4.98	0	0
Oregano	14.3±0.96	12.30±0.55	8.18±4.78	9.00±0.99 (n=2)	0
Fir	15.81±1.88	13.99±2.22	10.00±1.98	10.04±0.42 (n=2)	0
Sunflower	13.90±0.93	12.43±1.70	9.67±1.08	0	0
Heath	14.32±2.21	12.30±2.07	8.74±5.1	9.4 (n=1)	0
Manuka	17.40	13.10	9.1	0	0
Artificial honey	6.4	0	0	0	0
Significance (One way ANOVA)	-	-	-	-	-

Table S6. Geometrical means of the antibacterial activity of the different honeys against *Klebsiella pneumoniae* subsp *pneumoniae* (1) assessed by the MIC method as crude sample, with the addition of catalase and protease.

Honey type	MIC95 values (% v/v)		
	Crude samples	Catalase addition	Protease addition

**Cotton**	7.43	12.5	10.51
**Arbutus**	7.18	10.88	12.5
**Chestnut**	8.25	21.76	10.51
**Thyme**	7.18	9.47	9.47
**Orange**	8.84	14.86	10.51
**Oregano**	6.25	8.84	8.84
**Fir**	5.13	6.90	6.90
**Sunflower**	9.92	19.84	12.5
**Heath**	8.25	10.88	14.36
**Manuka**	6.25	25	12.5
**Artificial honey**	-	F=4.380606	
		P=0.000448	

Table S7. Antibacterial activity (zone of inhibition in mm) of various concentrations of the different honeys against *Klebsiella pneumoniae* subsp *pneumoniae* (2) assessed by the well diffusion method.

Honey type	**Inhibition zone (in mm)**				
	**75%**	**50%**	**25%**	**12.5%**	**6.25%**
Cotton	15.48±2.44	13.73±1.45	11.58±0.39	9.05±0.91 (n=2)	0
Arbutus	15.44 ±1.84	13.96±1.13	12.12±1.10	10.03±0.12 (n=3)	0
Chestnut	14.64±1.64	13.46±1.80	12.13±1.87	9.77±0.81 (n=3)	9.1 (n=1)
Thyme	13.96±0.23	12.26±0.56	10.46±0.27	8.60±0.71 (n=2)	0
Orange	13.83±0.33	12.30±0.24	10.75±0.68	0	0
Oregano	14.10±0.66	11.83±1.04	9.53±0.60	8.10±0.328 (n=2)	0
Fir	15.50±1.17	13.12±1.21	10.39±1.16	9.27±0.06 (n=3)	7.4 (n=1)
Sunflower	14.43±0.59	11.60±0.91	8.93±0.93	0	0
Heath	15.08±1.75	12.06±1.31	9.40±1.91	8.7 (n=1)	0
Manuka	16.5	12.80	9.3	8.4	0
Artificial honey	6.4	0	0	0	0
Significance (One way ANOVA)	F=2.157406 P=0.045356	F=2.919213 P=0.00887	F=5.205593 P<0.001	-	-

Table S8. Geometrical means of the antibacterial activity of the different honeys against *Klebsiella pneumoniae* subsp *pneumoniae* (2) assessed by the MIC method as crude sample, with the addition of catalase and protease.

**Honey type**	**MIC_{95} values (% v/v)**		
	**Crude samples**	**Catalase addition**	**Protease addition**
**Cotton**	7.43	12.50	7.43
**Arbutus**	6.25	10.51	8.25
**Chestnut**	7.18	16.49	10.88
**Thyme**	9.47	10.88	12.50
**Orange**	8.84	35.35	12.50
**Oregano**	7.43	12.50	6.90
**Fir**	5.13	7.62	12.50
**Sunflower**	9.92	19.84	12.50
**Heath**	8.25	10.88	10.51

**Manuka**	6.25	25.00	12.50
**Artificial honey**	-	-	-
	-	F=8.053138<br>P<0.0001	-

Table S9. Geometrical means of the MICs values of the honeys' extracts by different solvents against *P. aeruginosa.*

Honey type	Solvent			
	**n-hexane**	**diethylether**	**chloroform**	**ethylacetate**
**Cotton**	11.18	2.5	10.62	3.15
**Arbutus**	5	1.65	9.36	3.59
**Chestnut**	9.21	4.51	8.80	3.97
**Thyme**	8.59	3.37	7.09	5
**Orange**	2.5	9.84	8.55	10
**Oregano**	8.41	2.1	6.12	4.45
**Fir**	3.71	1.38	2.5	3.36
**Sunflower**	9.09	3.96	0	4.54
**Heath**	9.31	3.74	7.07	2.66
STATISTICAL SIGNIFICANCE	**F=7.501293<br>P<0.001**	**F=5.758054<br>P<0.001**	**F=6.828794<br>P<0.001**	**F=2.92929<br>P=0.011617**

Table S10. Geometrical means of the MICs values of the honeys' extracts by different solvents against *K. pneumoniae* subsp *pneumoniae* (1).

Honey type	Solvent			
	**n-hexane**	**diethylether**	**chloroform**	**ethylacetate**
**Cotton**	8.25	0.99	0.88	2.5
**Arbutus**	5.42	0.79	1.65	1.25
**Chestnut**	2.5	1.05	1.49	1.25
**Thyme**	2.22	1.89	3.30	3.30
**Orange**	6.69	1.25	2.5	2.5
**Oregano**	1.77	0.74	1.50	2.10
**Fir**	1.91	0.67	1.03	2.26
**Sunflower**	3.54	1.57	3.15	1.98
**Heath**	2.66	0.50	1.49	3.15
**STATISTICAL SIGNIFICANCE**	**F=3.317438<br>P=0.005681**	**NS<br>P=0.461486**	**NS<br>P=0.0698**	**NS<br>P=0.113214**

Table S11. Geometrical means of the MICs of the honeys' extracts by different solvents against *E. cloacae* subsp *dissolvens.*

Honey type	Solvent			
	**n-hexane**	**diethylether**	**chloroform**	**ethylacetate**
**Cotton**	3.97	0.99	6.30	2.5
**Arbutus**	1.09	1.09	2.5	1.25
**Chestnut**	1.77	0.44	1.77	1.25
**Thyme**	2.18	0.88	4.72	1.25
**Orange**	5.15	1.25	5.72	2.97
**Oregano**	4.22	0.63	2.5	3.54
**Fir**	1.13	0.62	1.99	1.52
**Sunflower**	2.5	0.63	0.99	1.57

Heath	4.65	0.95	1.09	3.67
**STATISTICAL SIGNIFICANCE**	**F=4.75301 P<0.001**	**NS P=0.475181**	**NS P=0.193689**	**F=4.345257 P<0.001**

Table S12. Geometrical means of the MICs values of the honeys' extracts by different solvents against *K. pneumoniae* subsp *pneumoniae* (2).

**Honey type**	**Solvent**			
	**n-hexane**	**diethylether**	**chloroform**	**ethylacetate**
**Cotton**	1.25	0.25	3.54	1.25
**Arbutus**	1.89	0.63	1.89	1.44
**Chestnut**	1.25	0.63	1.25	1.25
**Thyme**	0.99	0.35	3.27	2.81
**Orange**	3.15	0.31	8.27	2.5
**Oregano**	2.97	0.26	7.28	2.10
**Fir**	1.38	0.46	2.5	2.5
**Sunflower**	1.25	0.79	7.77	3.15
**Heath**	1.65	2.18	4.55	2.87
**STATISTICAL SIGNIFICANCE**	**F=3.086028 P=0.012178**	**F=8.249112 P<0.001**	**F=5.99412 P<0.001**	**NS P=0.068691**
